# James Fleming

**Published:** 1875-03

**Authors:** 


James Fleming, D. D. S., M. D., a native of Washington County, Pa., was for many years the leading dentist of Harrisburg, in the same State, where he died on the 30th of January, 1875. Dr. Fleming graduated in medicine at the Jefferson Medical School, Philadelphia, in 1838, received the degree of D. D. S. from the Baltimore College of Dental Surgery in 1851, and for more than thirty years practiced dentistry in Harrisburg, where he enjoyed the reputation of being a good operator and consciencious, upright gentleman, respected by all who knew him, and whose death will be long lamented by his many friends.

